# Impact of endodontic case difficulty on operating time of single visit nonsurgical endodontic treatment under general anesthesia

**DOI:** 10.1186/s12903-021-01586-0

**Published:** 2021-05-03

**Authors:** Shin Hye Chung, Juhea Chang

**Affiliations:** 1 Department of Dental Biomaterials Science, School of Dentistry and Dental Research Institute, Seoul National University, 101, Daehak-ro, Jongno-gu, Seoul, 03080 Republic of Korea; 2 National Dental Care Center for Persons with Special Needs, Seoul National University Dental Hospital, 101, Daehak-ro, Jongno-gu, Seoul, 03080 Republic of Korea

**Keywords:** Endodontic case difficulty assessment, General anesthesia, Operating time, Root canal morphology, Single-visit endodontic treatment

## Abstract

**Background:**

A Case Difficulty Assessment Form was designed for use in endodontic curricula, and to assist practitioners with treatment planning, referral and recording. The aim of this study was to determine how endodontic case difficulty factors influence the operating time of single-visit nonsurgical endodontic treatments under general anesthesia.

**Methods:**

Data on 198 single-visit endodontic treatments (80 anterior teeth, 43 premolars, and 75 molars) performed under general anesthesia by a specialized practitioner were obtained from 119 special needs patients (mean [SD] age = 30.7 [14.7] years). Total duration of operation was analyzed with relation to demographic and dental factors and American Association of Endodontists (AAE) Case Difficulty Assessment factors. Mann–Whitney U test, t-test, and Kruskal–Wallis test were used to assess relationships between operating time and confounding factors (p < 0.05).

**Results:**

High difficulty cases required significantly longer time to complete operations than treatments of minimal-to-moderate difficulty regardless of tooth type (p < 0.05). Demographic factors of the patients rarely influenced operating time length. Among variables included in the AAE Case Difficulty Assessment Form, tooth position, crown morphology, root morphology, canal appearance, and periodontal condition were significantly associated with increased operating time (p < 0.05).

**Conclusions:**

A higher level of case difficulty contributed to increased duration of endodontic treatment under general anesthesia indicating that Endodontic Case Difficulty Assessment Form is useful for predicting the duration of nonsurgical endodontic treatment. Among many factors, complicated anatomic features of the treated teeth increased case complexity and extended operating time.

## Background

Endodontic case difficulty levels were originally introduced to provide guidelines for general dental practitioners to assess the cases with complexities and refer the patients accordingly to specialists [1]. Another purpose was to evaluate the difficulty of cases and to select adequate cases for undergraduate teaching. There are several case difficulty assessment forms based on pre-treatment clinical findings and radiographic examination. The American Association of Endodontists (AAE) Case Difficulty Assessment Form was designed for use in endodontic curricula, and to assist practitioners with endodontic treatment planning, referral decisions and record keeping [2, 3]. It has three parts; general health consideration, diagnosis and treatment condition, and additional contributing factors such as trauma, previous treatment, and periodontal disease [4]. All factors are considered for assessment with final assignment to three difficulty levels: minimal, moderate, and high. In previous studies, difficulty levels based on AAE case difficulty assessment were related to quality outcomes [1] and procedural risks [5] of nonsurgical endodontic treatments performed by dental students and general practitioners.

Dental treatments are based on resources, as in other surgical fields. Predicting operating time is critical for organizing clinical resources such as operating space, staff, and facilities. Therefore, time management and decision making is an essential skill for practitioners to manage subsequent events with smoothness [6]. Time issues in dental practices are critical, despite being difficult to analyze. Many complexities exist, correlated with time and confounding factors, because of the diversity of practitioners’ skill and experience, clinical environments, and patients’ individual conditions. Nevertheless, endodontic operating time has been used as a useful outcome measure to evaluate the efficiency of clinical tools such as preclinical tooth models [7], magnifying loupe [8], and rotary file systems [9].

As for case complexity for dental specialties, endodontic cases have a particularly large variation in complexity, affecting treatment outcomes. Completion of difficult cases is expected to require more time and effort, even for specialized practitioners. To determine the contributing factors to increased operating time, restricting clinical diversities and analyzing cases treated in uniform circumstance is essential. An ideal condition can be obtained in hospital dentistry, where single-visit endodontic treatments are completed under general anesthesia (GA) by a single practitioner without an intermittence [10].

The aims of this study were to first determine if the level of AAE Endodontic Case Difficulty was associated with operating time for single-visit nonsurgical endodontic treatment under GA. Second, we determined what factors from the case difficulty assessment form significantly contributed to extending the time to complete treatment. The hypothesis tested in this study was that endodontic case difficulty would not affect the operating time of single-visit nonsurgical endodontic treatment under GA.

## Methods

### Case selection

Data on 198 endodontic cases were obtained for 119 patients (44 females and 75 males, mean age = 30.7, standard deviation [SD] = 14.7) who visited the Special Care Clinic, Seoul National University Dental Hospital for dental treatment from January 2016 through July 2019. Each treatment was performed in a single appointment under GA by one practitioner who was a board-certified specialist in endodontics and restorative dentistry (J.C.). Study inclusion criteria were: (1) single-visit nonsurgical endodontic treatment performed for at least one permanent tooth, and (2) GA administrated due to lack of cooperation resulting from patient’s intellectual or cognitive disabilities. The Seoul National University Dental Hospital Institutional Review Board approved this study (IRB No. CRI18005).

### Treatment procedures and time measurement

Endodontic procedures were described in previously published articles by the authors [10, 11]. After general anesthesia was administered by nasal intubation, digital intraoral radiographies were taken on the teeth (Schick CDR, Sirona, Long Island, NY, USA). Time recorded for preoperative radiography was the operation onset time. After access preparation with rubber dam isolation, working length was determined with an electronic apex locator (RootZX, J Morita, Irvine, CA, USA) and/or radiography. Root canals were enlarged and shaped with engine-driven instruments (Protaper Universal, Dentsply Maillefer, Tulsa, OK, USA) and stainless-steel K-files (Diadent, Cheongju, Korea). Canals were irrigated with 2.6% sodium hypochlorite. In cases with other restorative and/or periodontal treatments during the same GA procedure, canals were filled with irrigating solution and the access cavity was sealed with sterile cotton pellets and temporary filling material (Caviton, GC cooperation, Tokyo, Japan) for extended disinfection. This additional soaking time was excluded from time assessments. After radiographic confirmation of master cone fit, the intracanal space was filled with gutta percha cones (DiaDent, Cheongju, Korea) and root canal sealer (AH 26, Dentsply Detrey, Konstanz, Germany) with continuous wave of condensation at 200°C. The remaining space was backfilled with gutta percha using B&L Alpha II and Beta (B&L Biotech, Ansan, South Korea). After obturation of the canal space, intraoral radiography was taken to confirm outcomes. The time for postoperative radiography was regarded as the treatment endpoint. Total operation duration was calculated in minutes by subtracting time points for postoperative and preoperative radiography. Afterwards, intra-orifice and chamber spaces were filled with adhesive resin (SE Bond, Kuraray, Tokyo, Japan) and flowable composite resin (Tetric N-Flow, Ivoclar Vivadent, Schaan, Liechtenstein) and the access cavity was restored with composite resin (Tetric N-Ceram, Ivoclar Vivadent).

### Endodontic case difficulty assessment

The level for each factor in the AAE Case Difficulty Assessment Form was assigned by the same practitioner based on preclinical documentation and radiography. Factors based on radiography (canal morphology, canal appearance, and apical resorption) were rated by the practitioner and an independent judge who is specialized in endodontics for more than 10 years. Treatment outcomes based on postoperative radiography were rated for length and density of intracanal obturation by these two judges. In cases of disagreement on rating, reevaluation was performed until joint agreement. The consensus score for each factor was considered as the true value for statistical analysis. Difficulty levels were assessed in two ways: Model 1 was based on assessment of factors in all three categories (A. patient considerations, B. diagnostic and treatment considerations, and C. additional considerations), and Model 2 was based on only the latter two (B and C).

### Statistical analysis

Comparisons of operating time for patient demographic and dental factors were performed using Mann-Whitney U and Kruskal–Wallis tests. Relationships among mean operating time and case difficulty levels were analyzed using Kruskal–Wallis and *t*-tests. Multivariate linear regression was used to compare time and difficulty levels after adjusting for potential confounding factors. SAS version 23.0 (SAS Institute Inc, Cary, NC, USA) was used for analysis with alpha level 0.05.

## Results

The mean (SD) operating times were 42.2 (12.2) minutes for anterior teeth, 51.5 (19.3) for premolars, and 81.9 (19.5) for molars. Tooth type was a significant factor affecting operating time (p < 0.05, Table 1). Cases with unacceptable canal filling length and density required a significantly longer operating time than other cases (p < 0.05). For patient consideration in the AAE Case Difficulty Assessment, the factor of emergency condition significantly influenced operating time (p < 0.05, Table 2). For diagnosis and treatment considerations, radiographic difficulties and tooth position in the arch were factors significantly related to operating time (p < 0.05). Crown morphology complicated by restoration, deviation, or destruction and reduced or indistinct canal appearance were also significant factors (p < 0.05). For additional considerations, periodontal disease was significantly associated with operating time (p < 0.05). For case difficulty factors based on radiography, interjudge agreement for crown morphology was 0.98, for root morphology 0.96, for canal appearance 0.95, and for apical resorption of treated teeth 0.86. For treatment outcomes, interjudge agreement for filling length was for 0.81 and for density 1.00. Demographic factors of patients such as gender, age, disability type, medication, caregiver type, meal type, oral hygiene maintenance, and cooperation level were not significantly associated with operating time (data not shown).

**Table 1:** Operating time in minutes for single-visit nonsurgical endodontic treatment under general anesthesia (GA) related to the dental factors

Factors	N (%)	Time (mean±SD)	*P* value
Reason for endodontic treatment			
Caries	141 (71.2)	60.3±26. 7	0.65
Others	57 (28.8)	56.6±18.7	
Pulpal condition			
Nonvital	135 (68.2)	59.4±23.4	0.54
Vital	63 (31.8)	58.9±27.4	
Tooth type			
Anterior	80 (40.4)	42.2±12.2	< 0.001
Premolar	43 (21.7)	51.5±19.3	
Molar	75 (37.9)	81.9±19.5	
Canal filling length			
Acceptable	190 (96.0)	58.0±23.8	0.01
Unacceptable	8 (4.0)	87.9±29.4	
Canal filling density			
Acceptable	194 (98.0)	58.6±24.3	0.01
Unacceptable	4 (2.0)	92.5±20.2

**Table 2:** Operating time in minutes for single-visit nonsurgical endodontic treatment under GA related to endodontic case difficulty factors

Factors	N (%)	Time (mean±SD)	*P* value
*A. Patient considerations*			
Medical history			
ASA 1	74 (37.4)	58.8±24.4	0.75
ASA 2 or ASA 3	124 (62.6)	59.5±24.9	
Anesthesia			
No history of anesthesia problems	196 (99.0)	58.8±24.4	0.05
Vasoconstrictor intolerance	2 (1.0)	100.0±28.3	
Patient disposition			
Cooperation with both clinical and radiographic examination	40 (20.2)	66.9±26..5	0.06
Cooperation with either clinical or radiographic examination	134 (67.7)	57.0±23.2	
Cooperation with none of examination	24 (12.1)	59.0±28.1	
Ability to open mouth			
No limitation	188 (94.9)	59.5±24.4	0.61
Limitation	10 (5.1)	54.6±30.9	
Gag reflex			
None	–	–	–
Occasional or extreme	–	–	
Emergency condition			
Minimum pain or swelling	189 (95.5)	58.3±24.3	0.02
Moderate pain or swelling	9 (4.5)	78.9±26.6	
*B. Diagnosis and treatment considerations*			
Diagnosis			
Signs and symptoms consistent with pulpal and periapical conditions	181 (91.4)	59.0±24.7	0.67
Extensive differential diagnosis	17 (8.6)	61.5±24.6	
Radiographic difficulties			
Minimal	190 (96.0)	58.1±24.4	< 0.01
Moderate	8 (4.0)	87.0±13.8	
Tooth position			
Anterior/premolar Slight inclination or rotation < 10°	123 (62.1)	45.6±15.9	< 0.01
1st, 2nd, or 3rd molar Moderate or extreme inclination or rotation ≥ 10°	75 (37.9)	81.6±19.7	
Isolation			
Routine rubber dam placement	172 (86.9)	58.7±24.3	0.40
Pretreatment modification	26 (13.1)	63.1±27.1	
Crown morphology			
None of complication	103 (52.0)	51.3±21.6	<0.01
Moderate or severe complication	95 (48.0)	67.8±25.0	
Canal and Root morphology			
Slight or no curvature < 10° Closed apex (< 1 mm in diameter)	117 (59.1)	47.3±18.3	< 0.01
Moderate or extreme curvature ≥ 10° Open apex (≥ 1 mm)	81 (40.9)	76.5±22.5	
Canal appearance			
Not reduced	137 (69.2)	52.7±22.4	< 0.01
Reduced or indistinct	61 (30.8)	74.0±23.2	
Apical resorption			
None	130 (65.7)	58.0±25.0	0.20
Minimal or extensive	68 (34.3)	61.7±24.2	
*C. Additional considerations*			
Trauma history			
None	182 (91.9)	60.2±25.3	0.10
Fracture or luxation	16 (8.1)	48.7±10.8	
Endo treatment history			
None	181 (91.4)	59.7±25.1	0.45
Previous access	17 (8.6)	54.0±19.3	
Perio-Endo condition			
None or mild periodontal disease	183 (92.4)	57.7±24.2	<0.01
Moderate periodontal disease	15 (7.6)	77.6±23.9	

In Model 1 assessment with all categories included, case difficulty levels were significantly related to operating time for anteriors and molars (p < 0.05, Table 3). In Model 2 assessment with patient considerations excluded, difficulty levels were significantly related to time for all tooth types (p < 0.05, Table 4). In multivariate analysis for relationships between time and case difficulty, significant predictor variables were tooth position, crown morphology, and canal morphology (p < 0.05, Table 5).

**Table 3:** Operating time in minutes related to case difficulty levels (Model 1) with inclusion of all factors (categories A, B, and C)

Tooth types	Case difficulty levels (Model 1^¶^)						*P* value^§^
	Minimal		Moderate		High		
	N	Time (min)*	N	Time (mean±SD)	N	Time (mean±SD)	
Anterior	2	35.0 ± 7.1	55	40.6 ± 12.5	23	46.6 ± 10.9	0.04
Premolar	0	–	27	47.1 ± 12.5	16	59.0 ± 26.0	0.10
Molar	0	–	28	70.4 ± 15.3	47	88.7 ± 18.6	< 0.00
Total	2	35.0 ± 7.1	110	49.8 ± 18.1	86	71.9 ± 26.4	< 0.00

^¶^Endodontic case difficulty level was set with inclusion of the factors in three categories (A. patient considerations, B. diagnosis and treatment considerations, and C. additional considerations)

^§^Groups of minimal and moderate levels were combined for analysis

**Table 4:** Operating time in minutes according to the case difficulty levels (Model 2) with inclusion of dental factors (categories B and C)

Tooth types	Case difficulty levels (Model 2^¶^)						*P* values
	Minimal		Moderate		High		
	N	Time (mean±SD)*	N	Time (mean±SD)	N	Time (mean±SD)	
Anterior	18	33.7±10.0	51	44.0±12.0	11	47.6±10.0	0.00
Premolar	16	42.3±10.2	21	54.2±22.0	6	66.8±17.2	0.01
Molar	0	–	33	70.8±15.0	42	90.6±18.3	< 0.00
Total	34	37.7±10.8	105	54.5±19.2	59	80.2±24.0	< 0.00

^¶^Endodontic case difficulty level was set with inclusion of the factors in two categories (B. diagnosis and treatment considerations and C. additional considerations)

**Table 5:** Multivariate analysis for relationships among operation time and the endodontic case difficulty factors

Factors	*β*	*P* value	95% CI	R^2^
Emergency condition				
Minimum pain or swelling (Ref)	4.24	0.48	−7.49, 15.98	
Moderate pain or swelling				
Radiographic difficulties				
Minimal (Ref)	10.44	0.08	−1.44, 22.32	
Moderate				
Tooth position				
None of slight inclination or rotation < 10° (Ref)	24.57	< 0.01	17.62, 31.52	
1st, 2nd, or 3rd molar, or rotation or inclination ≥ 10°				
Crown morphology				
None of complication (Ref)	10.93	< 0.01	6.09, 15.76	0.59
Moderate or severe complication				
Canal and Root morphology				
Slight or no curvature < 10°, Closed apex (Ref)	9.26	< 0.01	2.93, 15.58	
Moderate or extreme curvature ≥ 10°, Open apex				
Canal appearance				
Not reduced (Ref)	4.51	0.11	−1.07, 10.09	
Visible but reduced or indistinct or not visible				
Perio-Endo condition				
None or mild periodontal disease (Ref)	2.46	0.59	−6.50, 11.42	
Moderate periodontal disease				

## Discussion

This study evaluated how the factors from case difficulty assessment by AAE were associated with operating time for single-visit nonsurgical endodontic treatments under GA. Among many clinical variables contributing to case difficulty, anatomical complexities of teeth were the main contributors to increasing operating time. So, our null hypothesis was rejected.

Operating time is a critical issue for practitioners, administrators, and third-party payers [12]. Previous studies determined factors affecting surgical duration to enhance the flow of operating rooms and working staff. Efficient scheduling can decrease expenditures by enhancing utilization of resources and minimizing overhead. In dentistry, a single practitioner mainly engages in an entire procedure with assisting staff at dental units. Therefore, a relatively smaller number of variables affect operating circumstances. From this point, case complexities can be a principal focus on time organization, staff stress, and financial management.

The administration of GA was inevitable for the study population, who could not comply with treatment because of intellectual and cognitive limitations. The authors had previously published studies on the quality assessment of endodontic treatment under GA [10, 11]. These studies validated the outcome assessments of single-visit endodontic and restorative treatments for patients with special needs and supported the treatment regimen under GA be encouraged. However, cost issues are inevitable in treatment planning and decision-making for special needs patients. Therefore, we attempted to introduce clinical time measurement as a treatment outcome, one of the most critical considerations in GA settings. In this study, the 198 cases of nonsurgical endodontic treatment of minimal to moderate or high difficulty were proportional (111 vs. 80). Across procedures, time-deciding factors were largely dependent on the case *per se* and minimally affected by other background issues. Furthermore, all treatments were completed by a single specialist who was accustomed to this clinical setting. Total treatment duration was relatively invariant: the SDs for mean operating time did not exceed 12 min for anterior teeth and 19 min for posterior teeth (Table 1). Nevertheless, high-difficulty cases required almost 150% more time to complete procedures than moderate-difficulty cases (Tables 3, 4).

Endodontic cases vary in complexity among dental practices. Many case-difficulty assessment forms have attempted to evaluate treatment complexity for practitioners and educators. On AAE assessment forms, a point system was introduced to assign a graded score according to the difficulty level. However, the weight of the value for an individual item in each category needs to be considered, since some items are more influenced by practitioner competence than others. In this study, complicated tooth anatomies were significant contributors to prolonged operations. Our multivariate analysis showed that high difficulty factors characterized by deformed crown shapes, deviated canal curvature, and/or indistinct canal path were strong predictors of increased time (Table 5). In a study of third molar extraction, anatomic variables were largely correlated with extraction time among many clinical factors that surgeons considered particularly important [13]. Patient demographics were minimally associated with operating difficulty, in accordance with our results. Often, surgeon stress level in operating rooms is quantified by numerical systems related to case complexity. It is encouraging that the subjective assessment of practitioner burden and the objective assessment of operating time correlated for more refined validation of case difficulty assessment.

Endodontic case difficulty levels have been used to evaluate quality outcomes for educational and referral purposes. For high difficulty cases, a higher number of mishaps and treatment visits occurred in undergraduate clinics [5] and more unacceptable outcomes were obtained from general practitioners [1]. In this study, the challenging conditions of difficult cases influenced operating duration and treatment outcomes, even for a well-experienced specialist. All cases with unacceptable levels of canal filling length and density belonged to the severe-difficulty category (data not shown) and required more than 150% of the time of cases with acceptable outcomes. This result showed that fulfillment of difficult cases was hard to achieve, although significant time and efforts were spent on completing treatment.

Among the factors of patient considerations included in Model 1 assessment, ability of mouth opening was only relevant in treatment under GA. Even using a mouth prop during procedures under GA, limited intraoral spaces impose difficulties on inserting and maneuvering intracanal instruments. However, other factors of patient considerations such as incompetent anesthesia, anxiousness, and gag reflex, did not affect procedural performance under GA administration. Moreover, of our study population, 63% had systemic disease, assigned with the American Society of Anesthesiologists (ASA) classification 2 or 3. Therefore, these cases were automatically subjected to the moderate (ASA 2) or high-difficulty category (ASA 3), regardless of the tooth conditions. We attempted to refine the assessment tool, so we analyzed it using Model 2 assessment that included more representative items in conventional practices (diagnostic and treatment considerations, and additional considerations). We finally obtained 34 minimal, 105 moderate, and 59 high-difficulty cases from Model 2 assessment, and a higher case difficulty resulted in a longer procedural duration for all types of teeth (Table 4). This result implied that in a conventional clinical setting, where relatively healthier patients are treated, tooth-related difficulty variables would have a clearer impact on operating duration. In another way, clinical factors such as pain control, anxiety, and mouth opening, which were minimally influential on treatment under GA, would be more related to operating time in treatment under local anesthesia. Although there are limitations in extrapolating this study’s outcomes to time prediction in a conventional clinical situation, it is clear that operating time will increase with the level of case difficulty regardless of clinical circumstances.

Conventionally, case complexity is considered in operating rooms from two perspectives, resource allocation and surgeon load. Risk and difficulty of endodontic treatment can also be evaluated by these aspects; objective measures of time spent and subjective measures of stress load. Therefore, in a future study, assessment of practitioner burden for difficult cases should be considered using time and stress measures [14]. Eventually, more advanced assessment tools can be enhanced for referral and education, furthermore, for other options such as financial reward and legal justification.

## Conclusions

Higher level AAE endodontic case difficulty was significantly influenced time spent completing single-visit nonsurgical endodontic treatments. Anatomic complexities of crown and root were the main contributors to extended operating duration.

## Data Availability

The datasets used and/or analysed during the current study are available from the corresponding author on reasonable request.
